# Role of the Melanocortin System in Gonadal Steroidogenesis of Zebrafish

**DOI:** 10.3390/ani12202737

**Published:** 2022-10-12

**Authors:** Sandra Navarro, Diego Crespo, Rüdiger W. Schulz, Wei Ge, Josep Rotllant, José Miguel Cerdá-Reverter, Ana Rocha

**Affiliations:** 1 Centro de Investigación en Recursos Naturales y Sustentabilidad (CIRENYS), Universidad Bernardo O’Higgins, Avenida Viel 1497, Santiago 8370993, Chile; 2 Reproduction and Developmental Biology Group, Institute of Marine Research, NO-5817 Bergen, Norway; 3 Reproductive Biology Group, Department Biology, Division Developmental Biology, Science Faculty, Utrecht University, 3584 CH Utrecht, The Netherlands; 4 Department of Biomedical Sciences and Centre of Reproduction, Development and Aging (CRDA), Faculty of Health Sciences, University of Macau, Taipa, Macau 999078, China; 5 Instituto de Investigaciones Marinas, Consejo Superior de Investigaciones Científicas (IIM-CSIC), 36208 Vigo, Spain; 6 Control of Food Intake Group, Department of Fish Physiolgy and Biotechnology, Institute of Aquaculture de Torre de la Sal (IATS-CSIC), 12595 Castellón, Spain; 7 Centro Interdisciplinar de Investigação Marinha e Ambiental (CIIMAR), Terminal de Cruzeiros do Porto de Leixões, 4450-208 Matosinhos, Portugal

**Keywords:** POMC, MSH, agouti-signaling protein, ASIP, ovarian follicles, spermatogenesis, reproduction, fish

## Abstract

**Simple Summary:**

Control of reproduction in both males and females is complex, and a number of hormonal systems translate internal and/or external information to the hypothalamic-pituitary-gonadal (HPG) system. The control of reproduction integrates this high-energy demanding event to the internal (stored energy availability) and external (resources availability for progeny) conditions, thus increasing environmental fitting and offspring survival. In this paper, we describe new effects of the melanocortin system on gonadal physiology. Both adrenocorticotropic hormone (ACTH) and melanocyte-stimulating hormones (MSHs) modulate gonadal steroid secretion working throughout specific melanocortin receptors expressed in different gonadal cell types. The inhibitory effects of ACTH on gonadotropin-stimulated estradiol secretion seems to be related to the deleterious effects of stress on the female reproductive axis. On the contrary, the physiological involvement of MSH peptides on ovarian gametogenesis as well as the stimulatory effects of ACTH on testicular testosterone production remain unknown and further studies are required to understand melanocortin role on gonadal physiology.

**Abstract:**

In teleost, as in other vertebrates, stress affects reproduction. A key component of the stress response is the pituitary secretion of the adrenocorticotropic hormone (ACTH), which binds to the melanocortin 2 receptor (MC2R) in the adrenal glands and activates cortisol biosynthesis. In zebrafish, Mc2r was identified in male and female gonads, while ACTH has been shown to have a physiological role in modulating reproductive activity. In this study, the hypothesis that other melanocortins may also affect how the zebrafish gonadal function is explored, specifically steroid biosynthesis, given the presence of members of the melanocortin signaling system in zebrafish gonads. Using cell culture, expression analysis, and cellular localization of gene expression, our new observations demonstrated that melanocortin receptors, accessory proteins, antagonists, and agonists are expressed in both the ovary and testis of zebrafish (*n* = 4 each sex). Moreover, melanocortin peptides modulate both basal and gonadotropin-stimulated steroid release from zebrafish gonads (*n* = 15 for males and *n* = 50 for females). In situ hybridization in ovaries (*n* = 3) of zebrafish showed *mc1r* and *mc4r* in follicular cells and adjacent to cortical alveoli in the ooplasm of previtellogenic and vitellogenic oocytes. In zebrafish testes (*n* = 3), *mc4r* and *mc1r* were detected exclusively in germ cells, specifically in spermatogonia and spermatocytes. Our results suggest that melanocortins are, directly or indirectly, involved in the endocrine control of vitellogenesis in females, through modulation of estradiol synthesis via autocrine or paracrine actions in zebrafish ovaries. Adult zebrafish testes were sensitive to low doses of ACTH, eliciting testosterone production, which indicates a potential role of this peptide as a paracrine regulator of testicular function.

## 1. Introduction

The melanocortin neuroendocrine system is one of the most complex signaling systems in vertebrates. In addition to the melanocortin peptides encoded in the common precursor proopiomelanocortin (POMC), the system includes two endogenous antagonists that can also function as inverse agonists, agouti-signaling protein (ASIP) and agouti-related protein (AGRP) [1]. Melanocortin agonists, such as melanocyte-stimulating hormones (α-, β- and γ-MSH) and adrenocorticotropic hormone (ACTH), signal through five melanocortin receptors (Mc1r-Mc5r) which exhibit distinct expression domains and pharmacological profiles [2]. Mc2r is specific for ACTH, whereas the MSHs bind to the remaining four Mcrs, with Mc1R and Mc3R displaying the highest affinity for α-MSH and γ-MSH, respectively. Furthermore, some of these receptors require the participation of the melanocortin receptor accessory proteins (Mraps) to either reach its functional expression, such as Mc2r [3], or to fine tune its pharmacological profile, such as Mc4r [4]. This complexity is even higher in teleost fish as the genome of the teleost antecessor doubled, thus resulting in an expansion of the peptide/receptor system. Therefore, teleost fish display two or more POMC paralogue genes [5] yet also additional copies of *asip* and *agrp* [2], *mraps* [6] and *mcr* genes [2].

Melanocortin signaling plays a role in regulating multiple key physiological processes for animal survival, yet its involvement in the control of tissue pigmentation (Herraiz et al., 2021), regulation of energy balance [7,8], and control of corticosteroid synthesis during stress response [9] are the most researched aspects of this endocrine/neuroendocrine signaling system. Acute stressors elicit a significant increase in plasma ACTH released from pituitary corticotropic cells after POMC post-transcriptional specific processing by prohormone convertase 1 (PC1) in response to hypothalamic corticotrophin-releasing hormone (CRH) [10]. Plasma ACTH binds Mc2r-Mrap1 complex in the adrenal cortex promoting corticosteroid synthesis and secretion [11], which restores homeostasis following stress episodes, predominantly mobilizing fuel stores to make energy available for the increased metabolic demand [12]. ACTH can also transduce stress-related information, regardless of corticosteroid synthesis, by binding Mc4r-Mrap2 complex in the brain or various peripheral tissues [4,13].

Stress also has deleterious effects on reproductive processes of animals including fish [12]. Although the mechanisms are far from understood, the effects of stress are thought to be mediated by interactions between the hypothalamic-pituitary-adrenal axis (HPA), the HP-interrenal (HPI, the equivalent of fish HPA), and the HP-gonadal (HPG) axis. In fact, ACTH suppressed human chorionic gonadotropin (hCG)-stimulated estradiol (E2) synthesis in a dose-dependent manner in female zebrafish [14]. In this study, we report that not only ACTH is able to modulate the reproductive axis but that chemical MSH analogues and endogenous melanocortin antagonists also modulate gonadal steroid secretion. Morphological evidence is provided for the participation of the melanocortin system in the reproductive axis by studying receptor expression localization in both ovary and testis tissues. New evidence of a role for ACTH in androgen production in zebrafish testes is also presented.

## 2. Materials and Methods

### 2.1. Fish and Housing

Wild-type (WT) zebrafish (*Danio rerio*) stocks come from a background of TU (Tübingen, Nüsslein-Volhard Laboratory, Tübingen, Germany) strain. Adult zebrafish were maintained at the Institute of Aquaculture Torre de la Sal (IATS) facilities at a water temperature of 28 ± 2 °C and a 14 h/10 h light/dark cycle. Fish were fed a combination of freshly-hatched brine shrimp (*Artemia* sp. nauplii) and sera Vipan flake food (Sera, Heinsberg, Germany) three times a day until satiety. Experiments were performed in accordance with the Spanish (Royal Decree 53/2013) and European (2010/63/EU) legislation for the protection of animals used for experimentation. 

### 2.2. Quantitative Real-Time PCR (qPCR)

The expression of the key members of the melanocortin system was determined by quantitative PCR (qPCR) in whole gonads (ovary and testis). Total RNA was purified from adult zebrafish testes and ovaries (*n* = 4 for each sex) using TRI Reagent^®^ (Molecular Research Center, Inc., Cincinnati, OH, USA). The purified RNA was then treated with RQ1-DNAse (Promega Corp., Madison, WI, USA) in order to remove genomic DNA followed by ethanol precipitation. The RNA purity and concentration were verified by spectrophotometry (Nanodrop ND-2000 Spectrometer, Thermo Fisher Scientific, Waltham, MA, USA). One μg of total RNA from gonads was used for cDNA synthesis, which was performed with random hexamers (300 ng), dNTPs (2.5 mM of each dNTP) and 200 U of the SuperScript™ III Reverse Transcriptase (Invitrogen, Corp., Carlsbad, CA, USA) in 20 μL reactions. Reverse transcription conditions were: 25 °C for 10 min; 60 min at 50 °C for cDNA synthesis followed by 70 °C for 15 min to inactivate the reaction. The synthetized cDNA was stored at −20 °C for further use.

The expression of *mc2r*, *mc4r*, *mrap1*, *mrap2a,* and *mrap2b* in ovaries and testes were measured using TaqMan qPCR assays. For each 20 μL PCR, 1 μL of RT reaction was mixed with the corresponding amount of primers and probes (Table 1) in Abgene’s Absolute™ QPCR Mix (Thermo Scientific, Spain). Cycling conditions were 95 °C for 15 min, followed by 40 cycles at 95 °C for 15 s and 60 °C for 60 s. The relative mRNA levels of *mc1r*, *mc3r*, *mc5ra*, *mc5rb*, *pomca*, *pomcb*, *asip1*, *agrp1,* and *agrp2* were quantified by mixing 1 μL of cDNA template with specific primers (Table 1) and Abgene’s SYBR^®^ Green QPCR Master Mix (Thermo Scientific, Spain) in a total reaction volume of 15 μL. Thermal cycling conditions were 95 °C for 15 min, followed by 40 cycles at 95 °C for 15 s, a specific annealing temperature for 15 s and 72 °C for 15 s. Following completion of the amplification process, a melt curve was generated by increasing the temperature to 0.5 °C increments/10 s starting from 55 °C to 95.5 °C in order to verify product specificity. A no template control, using water instead of a cDNA sample, was included on each plate. Samples were run in duplicate on an CFX96™ (Bio-Rad Laboratories, Inc.), using 96 well optical plates and default settings.

Expression levels of the *18s rRNA* gene (Table 1) in 1:10,000 (ovary and testis)- or 1:1000 (follicular cells)-diluted cDNA samples (1 μL) were used as reference for data normalization. Data was recorded and analyzed by iCycler iQ™ software (version 3.0.6070). The 2^−(∆∆Ct)^ method [18] was used to calculate the relative fold gene expression.

### 2.3. Synthesis of Riboprobes

pGEM-T easy plasmids containing the full coding regions of the zebrafish *mcr1* and *mcr4* genes [4] were linearized with *Sal*I or *Apa*I and used to prepare antisense and sense riboprobes by in vitro transcription using T7 or SP6 RNA polymerase (Promega, Spain), respectively and digoxigenin (DIG)-labelled UTPs (Roche Diagnostics GmbH). Synthetized probes were treated with RQ1-DNAse-RNAse free (Promega, Spain) for 15 min at 37 °C to remove the DNA template. Ultimately, the probes were purified using Micro Bio-Spin Chromatography Columns (BioRad, Spain) and quantified in a Nanodrop 2000c spectrophotometer.

### 2.4. In Situ Hybridization

In situ hybridization experiments were carried out as previously described [4]. Adult fish (>90 days post fertilization (dpf)), with no signs of disease, were euthanized with an overdose of ethyl 3-aminobenzoate methanesulfonate (MS-222; 300–400 mg/L; Sigma-Aldrich, Spain) and their gonads carefully removed. Ovaries and testes from three fish of each sex were fixed in 4% paraformaldehyde (PAF)-phosphate buffer (PB; 0.1 M, pH 7.4) overnight at 4 ˚C, dehydrated, and embedded in Paraplast (Sherwood, St. Louis, MO, USA). Samples were cut in 5 µm sections, mounted on slides coated with 3-triethoxysilpropylamine and allowed to dry before being stored at 4 °C. Samples were used within a month. Prior to hybridization, sections were deparaffinized, re-hydrated, and post-fixed in 4% buffered PAF for 20 min. Slides were subsequently washed in PB 0.2 M (twice for 5 min) and treated with a Proteinase-K solution (20 µg/mL in 50 mM Tris-HCl, 5 mM EDTA, pH 8) for 5 min at room temperature (RT). This was followed by washing in 0.2 M PB and post fixed once again in 4% buffered PAF, subsequently rinsed in sterile water, and acetylated in a triethanolamine (0.1 M, pH 8)/acetic anhydride solution for 15 min in constant agitation. Sections were then dehydrated and dried at RT. Antisense or sense cRNA probes of mcr1 or mcr4 were preheated at 75 °C for 7 min and diluted in hybridization buffer [50% formamide, 300 mM NaCl, 20 mM Tris–HCl (pH 8), 5 mM EDTA (pH 8), 10% dextran sulphate, 1× Denhardt’s solution, and 0.5 µg/µL of yeast tRNA] at a concentration of 10 ng/µL. Sections were covered with 80–100 μL of hybridization solution, mounted with coverslips and incubated in a humidity chamber at 55 °C overnight. Slides were then washed in 5× saline sodium citrate buffer (SSC, 150 mM NaCl, 15 mM sodium citrate at pH 7) for 30 min at 55 °C to remove coverslips. Subsequently, they were rinsed in 2× SSC and 50% formamide for 30 min at 65 °C and immersed in NTE buffer (500 mM NaCl, 10 mM Tris–HCl, 5 mM EDTA, pH 7.5) three times for 10 min at 37 °C. Following the ribonuclease A treatment (2 μg/mL ribonuclease A in NTE) for 30 min at 37 °C, slides were incubated in NTE buffer for 10 min at 37 °C, once in 2× SSC and 50% formamide for 30 min at 65 °C, once in 2× SSC for 10 min at RT, twice in 0.1× SSC for 15 min at RT, and twice in buffer A (150 mM NaCl, 100 mM Tris-HCl, pH 7.5). In order to decrease the background, slides were previously incubated in blocking buffer (150 mM NaCl, 100 mM Tris-HCl, pH 7.5, 2% blocking reagent (Roche Diagnostic GmbH, Germany) for 30 min at RT. The slides were transferred to a humidified chamber, 1:1000 dilution of Fab fragments from an anti-digoxigenin antibody from sheep was added, conjugated with alkaline phosphatase (AP) in buffer A, and incubated at 4 °C overnight. The antibody was removed by washing twice in buffer B (100 mM Tris, 100 mM NaCl, 50 mM MgCl2, pH 9.5) for 10 min. A color substrate solution, composed of nitro blue tetrazolium chloride (NBT)/5-bromo-4-chloro-3-indolyl phosphate, toluidine salt (BCIP) (Roche Diagnostic GmbH, Germany) supplemented with levamisole (0.4 mg/mL NBT; 0.19 mg/mL BCIP; 1 mM levamisole) in buffer B was used as chromogen substrates for the detection of alkaline phosphatase. Color development was performed in the dark at RT, for 5 h. The reaction was concluded by washing the slides for 5 min with double distilled sterile water. Sections were mounted with a mount quick aqueous medium (Bio-Optica, Spain) and visualized on an Olympus BX41 microscope. Neighboring sections of the analyzed ovary and testis tissue were stained with haematoxylin-eosin and toluidine blue respectively, to distinguish the type of germ cells present in the in-situ hybridization signal.

### 2.5. Primary Cell Cultures

#### 2.5.1. Reagents

Lyophilized human chorionic gonadotropin (hCG, 50 I.U/mL) obtained from the urine of expectant women was purchased from Sigma-Aldrich (Merck KGaA, Darmstadt, Germany). Acetyl-(Nle4, Asp5, D-Phe7, Lys10)-cyclo-α-MSH (4–10) amide acetate salt (MTII) and Acetyl-(Nle4, Asp5, D-2-Nal7, Lys10)-cyclo- α-MSH (4–10) amide trifluoroacetate salt (SHU9119) were sourced from Bachem (Switzerland). Human adrenocorticotropic hormone (ACTH) (1–24) and human Agouti-related protein-(83-132)-NH2 (amidated carboxyl-terminal AGRP fragment) were purchased from Phoenix Pharmaceuticals Inc. (USA).

#### 2.5.2. Isolation of Testicular and Ovarian Cells

Adult WT males (*n* = 15) and females (*n* = 50) were euthanized by overdose of anesthesia (MS222, tricaine methane sulfonate; 300 mg/l). Cells from zebrafish testes were isolated according to the protocol published by [19] with modifications. Testes were carefully dissected under an Olympus SZX16 (Japan) microscope and retained in 0.5% bleach in Dulbecco’s phosphate-buffered saline with calcium and magnesium (DPBS^+^, Gibco ^TM^) for 2 min. Following a wash for 2 min in DPBS^+^, and with the help of fine surgical scissors, testis tissue was disaggregated in 1–1.5 mL of 0.15% collagenase/0.12% dispase in DPBS^+^ followed by a 2 h incubation at 28 °C in the same media. Pipetting was carried out every 20 min. Enzyme reaction was concluded by adding 12 mL of Leibovitz-15 medium (L-15, Gibco^TM^) supplemented with an antibiotic (1% penicillin-streptomycin) solution, 0.5% BSA and 10 mM HEPES (pH 7.4). Filtering the cell suspension through a sterile cell strainer (70 and 40 µm mesh size) allowed the removal of any large remaining tissue aggregates. Single-cell suspensions were centrifuged at 500 rpm for 10 min at RT. The supernatant containing the mature sperm fraction was discarded. The cell pellet composed of germ cells such as spermatogonia, spermatocytes and spermatids, and somatic cells, was re-suspended in 0.5–1 mL of fresh L-15 medium and cells were counted.

Gravid females were identified based on the presence of the genital papilla and larger abdomen. Ovaries were removed carefully and placed in a Petri dish containing RT L-15 medium used at 60% (pH 7.4) and supplemented with an antibiotic (1% penicillin-streptomycin) solution. The follicles were manually separated under a dissection microscope, the diameter was measured with an eyepiece micrometer, and subsequently the follicles were divided based on their size and vitellogenic state.

#### 2.5.3. In Vitro Treatments

Testicular cells were seeded on 96-well cell culture plates and incubated at 28 °C in a humidified air atmosphere to avoid evaporation. Cell density was 1 × 10^5^ cells/well. Cells were allowed to settle for approximately 24 h prior to cell stimulation (Kurita and Sakai, 2004). Prior to commencing the assay, fresh medium of 120 µL was added.

Healthy follicles at early vitellogenic stage (EV, ~0.35 mm) and mid vitellogenic stage (MV, ~0.45 mm) were selected, mixed, and placed in 24-well cell culture plates (50 follicles/well). Incubation was carried out according to the method published [20], at 28 °C in a humidified air atmosphere. Follicles were allowed to settle for 2 h and the medium was replaced by 200 µL of fresh 60% L-15. Testicular cells/ovarian follicles were treated with hCG and/or MTII, hACTH, SHU9119, and hASIP. Working dilutions, ranging from 1 × 10 ^−6^ to 1 × 10 ^−8^ M, were made fresh at the time of use. Following a 24 h incubation (males)/12 h (females) cultures were terminated by placing the plates on ice, carefully collecting and subsequently storing at −80 °C for further steroid analysis. The incubation periods of both testicular cells and ovarian follicles were determined according to preliminary time-course experiments (data not shown). To demonstrate replicability, the experiments were performed in triplicate and repeated at least three times.

#### 2.5.4. Steroid Release Analysis

In order to determine whether basal or hCG-stimulated gonadal steroid release was modulated by melanocortin peptides, the levels of testosterone (T) and estradiol (E2) were measured in the culture medium by means of ELISA kits following the manufacturer’s instructions (Neogen, USA). Both kits are non-species specific and were validated for culture medium samples by assessing parallelism of a serial dilution curve, run in duplicate with the standard curve. The serial dilution curve was obtained, by diluting in EIA buffer, a pool of culture medium samples, to make half dilutions (data not shown). The assays minimum detection limits were 2 and 20 pg/mL for T and E2, respectively. Steroid levels were expressed as a percentage (%) of the basal levels.

### 2.6. Data Analysis

Data are expressed as the mean ± SEM. Statistical treatment of the data was carried out with a GraphPad Prism version 9.1.1 with a significance level of 0.05. Statistical evaluation of the gene expression data was accomplished by ANOVA analysis thus followed by a post-hoc Sidak’s test. Statistical variations in steroid levels were analyzed by a one-way ANOVA test followed by Tukey’s post-hoc test. Gross deviations from the ANOVA assumptions of error normality and homoscedasticity were determined using Levene’s as well as the Kolmogorov-Smirnov test, respectively. Variations were considered statically significant when *p* < 0.05.

## 3. Results

### 3.1. Expression of the Melanocortin System in Zebrafish Gonads

**Figure 1:**
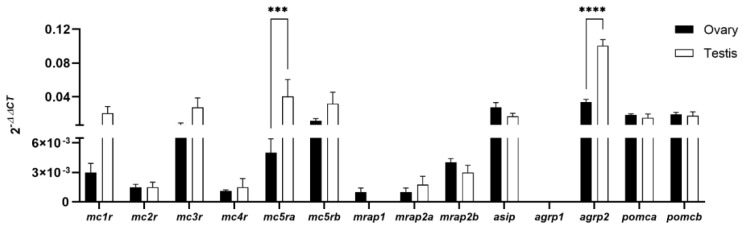
Relative expression levels of melanocortin-related genes in zebrafish ovaries and testes. Expression of the 18s rRNA gene was used as the reference gene for normalization of cDNA levels. Asterisk indicates significant variation after a two-way ANOVA analysis followed by Sidak’s multiple comparison test (*** *p* < 0.001, **** *p* < 0.0001). Data are expressed as mean ± SEM.

qPCR analyses revealed that melanocortin receptors, accessory proteins, antagonists, and agonists are expressed in both adult ovaries and testes of WT fish (Figure 1). The expression of both mc5r and agrp2 was significantly higher in male zebrafish whereas the expression levels of agrp1 and mrap1 (males) were under the detection limits.

### 3.2. Cellular Localization of mc1r and mc4r mRNA in Ovaries and Testes

**Figure 2:**
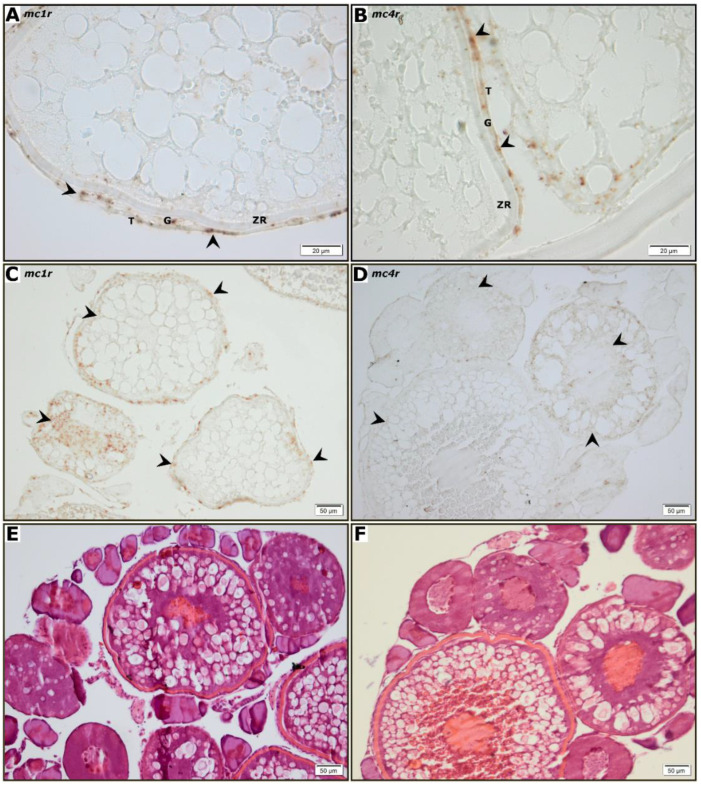
Localization of mcr1 and mcr4 transcripts expression in the zebrafish ovary by in situ hybridization. Panels (**A,B**) show mc1r and mc4r expression in the follicular cells, respectively. Panels (**C**,**D**) display mc1r and mc4r expression in the previtellogenic and vitellogenic follicles of the zebrafish ovary. (**E**,**F**) are representative tissue sections of (**C**,**D**) stained with haematoxylin-eosin, respectively. Signal was absent in the interstitial region.

**Figure 3:**
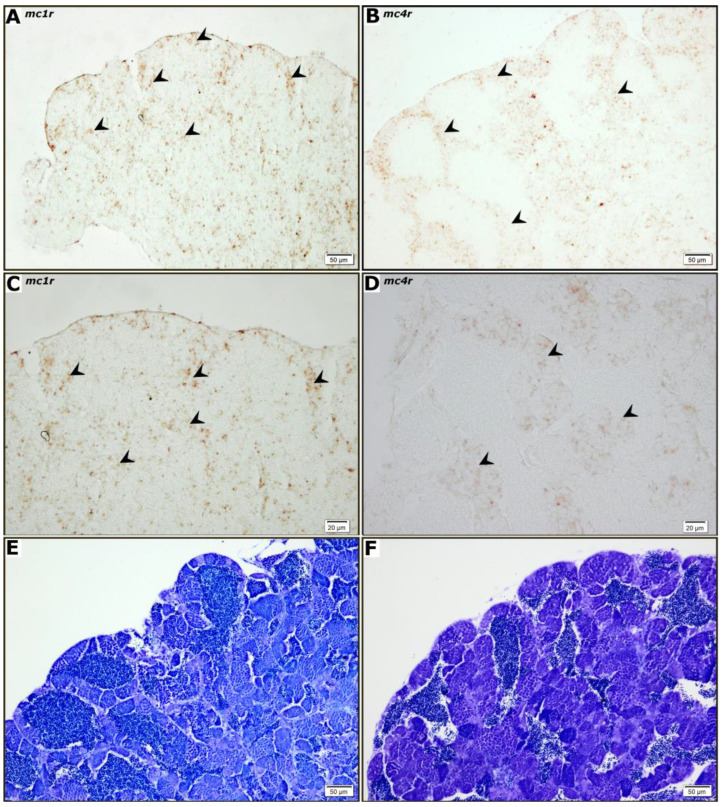
Localization of mcr1 and mcr4 transcripts expression in zebrafish testes by in situ hybridization. Panels (**A**,**B**) show mc1r and mc4r expression in germ cells, respectively. (**C**,**D**) are magnified views of (**A**,**B**) for detail. (**E**,**F**) are representative tissue sections of (**A**,**B**) stained with toluidine blue, respectively.

In order to investigate the cellular localization and maturation stage-dependent expression of *mc1r* and *mc4r*, an in situ hybridization study using ovary (Figure 2) and testis (Figure 3) sections of adult fish was carried out. No staining was observed in hybridization with sense cRNA probes for *mc1r* or *mc4r* (data not shown). For both receptors, a clear hybridization signal was observed in the follicular cells of ovarian follicles (Figure 2A,B). However, the cytoplasm of some previtellogenic (data not shown) and vitellogenic oocytes were also stained while the interstitial compartment showed no signal (Figure 2C,D). The staining obtained for *mc4r* mRNA (Figure 3B–D) was present mainly in larger germ cells arranged in groups located in the periphery of spermatogenic tubules. This pattern is compatible with spermatogenic cysts containing spermatogonia type A and B. The more prominent *mc1r* mRNA (Figure 3A–C) staining, however, seems to be present in part in germ cells, but also in some somatic cells, particularly in the periphery of the testis.

### 3.3. In Vitro Effects of Melanocortin Peptides on Basal and hCG-Induced Steroid Secretion by Zebrafish Gonadal Cells

The drugs used in the in vitro studies were previously tested on human embryonic kidney 293 cells transiently expressing zebrafish melanocortin receptors [17]. MTII is a potent universal agonist of melanocortin receptors. Not only can it activate all the zebrafish melanocortin receptors, but it also competes with human agouti-related protein-(83-132)-NH2 for the binding to the receptor when both drugs are present. SHU9119 is a potent synthetic Mc3r and Mc4r antagonist.

#### 3.3.1. Estradiol Secretion by Ovarian Follicles

**Figure 4:**
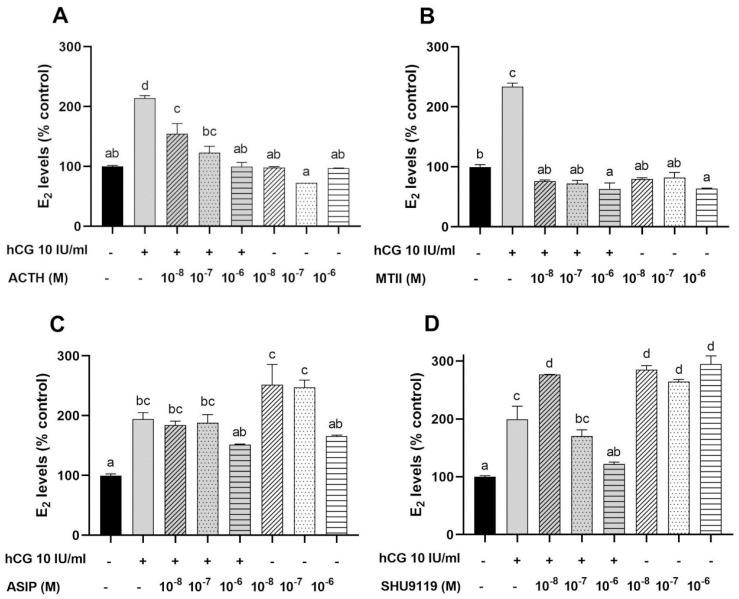
Effects of melanocortin peptides on basal and human chorionic gonadotropin (hCG)-induced estradiol (E2) secretion in previtellogenic and vitellogenic ovarian follicles of zebrafish. (**A**) Effects of ACTH, (**B**) MSH chemical analogues (MTII), (**C**) human ASIP, and (**D**) chemical antagonist (SHU9119). Experiments were performed in triplicate and repeated at least three times. Data is expressed as a percentage of the control (mean ± SEM). Different letters indicate significant variations after a one-way ANOVA analysis, followed by Tukey’s post-hoc test, *p* < 0.05.

As expected, the incubation of ovarian follicles with hCG (10 I.U./mL) resulted in a significant two-fold increase of E2 release into the culture medium in comparison to the basal control group (Figure 4). No significant differences in ACTH-(1–24) or MTII-induced E2 secretion were detected when exclusively used (Figure 4A,B). However, ACTH (1–24) and MTII significantly decreased hCG-stimulated E2 secretion in a dose-dependent manner (Figure 4A,B). With the lowest dose (10^−8^ M) of ACTH (1–24), a decrease in E2 release of ≈ 69% compared to hCG-only treatment was observed, while the highest dose (10^−6^ M) resulted in a ≈ 114% decrease of E2 in the media (Figure 4A). Similar results were observed when MTII was added to the media, yet with a higher potency, where the highest dose (10^−6^ M) decreased ≈ 170% the E2 production compared to an exclusive hCG treatment (Figure 4B). The effect of melanocortin antagonists was also studied. The presence of ASIP (10^−8^ M and 10^−7^ M) or SHU9119 (all tested concentrations) significantly enhanced follicular E2 release (≈ 150% and ≈ 180% above control levels, respectively) when exclusively used (Figure 4C,D). However, ASIP had no significant effect on hCG-induced E2 secretion while SHU9119, when used at a low concentration (10^−8^ M), significantly increases (≈ 77%) hCG-stimulated E2 production (Figure 4C,D).

#### 3.3.2. Testosterone Secretion by Testicular Cells

**Figure 5:**
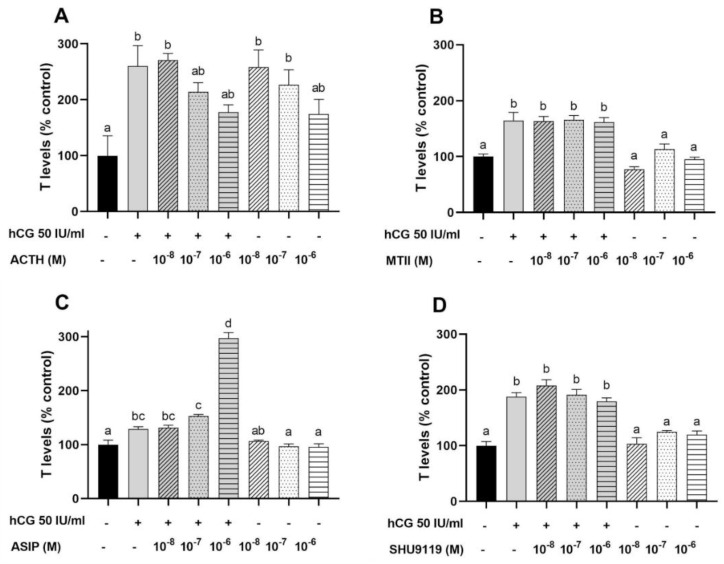
Effect of melanocortin peptides on basal and human chorionic gonadotropin (hCG)-induced testosterone (T) in primary cell cultures of dispersed testicular cells of zebrafish. (**A**) Effects of ACTH, (**B**) MSH chemical analogues (MTII), (**C**) human ASIP and (**D**) chemical antagonist (SHU9119). Experiments were performed in triplicate and repeated at least three times. Data is expressed as a percentage of the control (mean ± SEM). Different letters indicate significant variations after a one-way ANOVA analysis, followed by Tukey’s post-hoc test, *p* < 0.05.

As anticipated, increased production of T was detected in testicular cell cultures treated with hCG (50 I.U/mL) (Figure 5). Both agonists, ACTH (1–24) and MTII, as well as the antagonist SHU9119, did not modulate hCG-induced T production above basal levels (Figure 5A,B,D). At a high concentration (10^−6^ M), ASIP significantly potentiated (≈170% above the control) the hCG-induced T production, but had no effect when exclusively used (Figure 5C). At low doses (10^−8^ and 10^−7^ M), ACTH (1–24) increased ≈ 145% the release of T above the control group, while SHU9119 had no effect (Figure 5A,D).

## 4. Discussion

Previous studies have demonstrated that melanocortins can modulate gametogenesis in zebrafish. Specifically, in vitro studies showed that ACTH inhibits hGC-induced E2 secretion from ovarian follicles [14] providing mechanistic insights into the deleterious effects of stress on the female reproductive axis. Additional studies also demonstrated that *asip1* overexpression in a zebrafish transgenic model increased egg production yet reduced size and spawn frequency, once more suggesting that the melanocortin system may modulate the reproductive axis in females [5]. In fact, a role of Mc4r in the regulation of gonadal development and reproduction [21,22,23] of zebrafish and medaka (*Oryzias latipes*) has already been proven, yet involvement of the melanocortin system in the gonad function is poorly understood and no data is available regarding its role in male reproduction. In this study, we take a closer look at the function of the melanocortin system in zebrafish gametogenesis, firstly, by screening the expression of the key components of the system in zebrafish gonads in a single study, followed by examining the in vitro effects of agonists and antagonists on basal and hGC-stimulated steroid secretion in both ovaries and testes.

Results show that almost all melanocortin-related genes (except agrp1) were expressed in testes and ovaries with contrasting transcription levels. The presence of melanocortin receptors in vertebrate gonads has been recorded in other species including fish. A summary is supported in Table 2. In humans, MC1R protein was localized in Leydig and corpus luteum cells [24], the functions of which are related to the synthesis of steroids in males and females, respectively. In fish, *mc1r* transcripts are detected in adult organs, including the testes and ovaries, whereas in zebrafish, they were solely detected in the testes [2,25,26]. It was also shown that *mc2r* is expressed in both female and male gonads as previously reported in zebrafish [3,14]. A similar distribution was described in rainbow trout (*Oncorhynchus mykiss*) [27] and carp (*Cyprinus carpio*) [28], while in sea bass (*Dicentrarchus labrax*), *mcr2* was exclusively detected in the testes [29]. *mc3r* is expressed in zebrafish ovaries and testes as previously found in other fish species, including hibernating cavefish (*Onychostoma macrolepis*) [30], rainbow trout [31], and topmouth culter (*Culter alburnus*) [32]. In mammals, *Mc3r* is expressed in all the tissues of the hypothalamus-pituitary-gonad axis of adults [33] yet also in fetal testes of mice [34]. *Mc4r* is expressed principally in the central nervous system to regulate energy balance [35] in mammals yet transcripts have been detected in peripheral tissues as well as gonads of several species including zebrafish [4,36]. Moreover, *Mc5r* is expressed in a range of adult tissues found in mice with detectable levels in the testes [37]. In fetal mice, *Mc5r* was detected in spermatogonia and mesenchymal cells [38]. In birds, *Mc5r* is found in the ovaries and testes [39], while in rainbow trout, it is present in the anterior kidney, as well as the ovary [36]. In zebrafish, *mc5ra* and *mc5rb* are highly expressed in the ovary, brain, and gastrointestinal tract [40]. In the current study, both copies were detected in ovaries and testes.

Our further studies on melanocortin receptors expression by in situ hybridization, limited to *mc1r* and *mc4r*, showed that both receptors are expressed in the follicular cells and adjacent to cortical alveoli in the ooplasm of previtellogenic and vitellogenic zebrafish oocytes. In addition, *mc4r* and *mc1r* expression was detected in the germ cells of the testes, yet no signal was observed in somatic cells (Sertoli and Leydig). The distribution pattern obtained is similar to that of the *ddx4* gene, which codes for Vasa, considered a universal gene marker of germ cells [47]. It also resembles the distribution of *piwil1*, a type A and B spermatogonia marker and primary spermatocytes [48,49]. To a great degree, the evidence obtained suggests that the melanocortin effect is directly signaled through specific receptors expressed in both ovaries and testes, which further supports a role for the melanocortin system on gonadal physiology. Such effects could be mediated at an endocrine level by systemic hypophyseal melanocortins, yet also at a paracrine level, as α-MSH has also been localized in the follicular cells of previtellogenic, vitellogenic, and maturing oocytes, as well as in the cytoplasm of oogonia in fish [50,51,52]. In fact, the content of POMC-derived peptides varies according to the reproductive cycle, thus suggesting a role for such peptides in the direct modulation of ovarian activity [51]. Accordingly, it was demonstrated that both POMC paralogues are expressed in the testes and ovaries of zebrafish, thus suggesting a local processing and production of gonadal melanocortin peptides. Some melanocortin receptors require interaction with accessory proteins to either reach a functional expression (*mc2r*) [3] or fine tune the pharmacological profile (*mc4r*) [4]. ACTH signals principally through the above receptors, thus ACTH response inevitably requires the expression of MRAPs. Consequently, the expression of *mraps* in the gonadal tissues of zebrafish was studied showing that all three proteins (Mrap1, Mrap2a and Mrap2b) are expressed in the gonads of zebrafish, yet *mrap1* expression in males remains under the detection levels of qPCR. The gonadal expression of melanocortin accessory proteins has already been described in several vertebrate species, including fish [20,53,54]. Mc2r and Mc4r require interaction with both Mrap1 and Mrap2a to bind ACTH, respectively [4]. According to our results, both testes and ovaries retain the necessary molecular machinery to translate the ACTH programmed data throughout its key signaling complexes (Mc2r/Mrap1 and/or Mc4r/Mrpa2a). ACTH could convey stress-related data to the zebrafish ovary thus inhibiting hCG-stimulated E2 secretion [14] either independently of cortisol synthesis/secretion through MC4R/Mrap2a interaction [4,13], or stimulating cortisol synthesis/secretion through Mc2r/Mrap1 interaction [3]. The ACTH signaling pathway in the gonads, via Mc2r and/or Mc4r, has not been studied yet, but it has been recorded that fish gonads of both sexes are able to produce cortisol which supports the idea that glucocorticoids may play a role as a paracrine regulator of the gonadal physiology, e.g., by modulating transcript levels of genes involved in the gonadal function in zebrafish [55]. Although cortisol treatment had no effect on 11-ketotestosterone (11-KT) release, it stimulated spermatogonial proliferation in an androgen-independent manner as well as promoting meiosis and spermiogenesis by increasing the number of spermatozoa in the testes [55]. In contrast, a high concentration of cortisol (100 ng/mL) stimulated testicular production of 11-KT in vitro in Japanese eel (*Anguilla japonica)*, which suggests that the cortisol-induced spermatogonial proliferation might be mediated by androgens [56]. Similar findings were observed for pejerrey (*Odontesthes bonariensis*), where treatment with cortisol also increased 11-KT release from adult testis explants [57]. In fact, results from in vitro experiments fit into the concept that several tissue/cells participate in the organism’s androgen balance [58,59]. To a great degree, our expression studies provide evidence which proves that zebrafish gonads retain a gonadal hypophyseal-interrenal-like axis that may regulate gonadal cortisol synthesis/secretion in a paracrine manner. However, an endocrine role for the systemic melanocortins should not be ignored. This endocrine/paracrine role can be further modulated by the gonadal expression of endogenous antagonist due to the fact that both *asip* and *agrp2*, are expressed in both ovaries and testes. The expression of all melanocortin receptors in zebrafish ovaries and testes suggests that MSHs peptides could also have a role in the regulation of the zebrafish gonadal function. In fact, recent studies have demonstrated that in vivo treatment of cichlid fish (*Oreochromis mossambicus*) with α-MSH produces a reduction in the number of previtellogenic and vitellogenic follicles, thus decreasing the gonadosomatic index and increasing the rate of follicular atresia [60]. The mechanism seems to involve a significant reduction of the luteinizing hormone (LH) secretion mediated by α-MSH effects on the hypothalamic gonadotropin-releasing hormone (GnRH). Concerning black rockfish (*Sebastes schlegelii*), stimulation with homologous α- and β-MSH of ovaries was found to induce the expression of genes related to steroidogenesis [23].

The effects of both the melanocortin agonist and antagonist on steroid secretion in vitro were thus subsequently explored. As previously observed in zebrafish [14], ACTH had no effect on basal E2 secretion yet inhibited hCG-induced secretion in the previtellogenic and vitellogenic follicles. However, we can unprecedentedly demonstrate that MTII, a chemical agonist of MSH peptides, severely inhibits hCG-induced E2 secretion in the ovary. Such results are well in agreement with those showing that α-MSH treatment provokes a reduction in the number of ovarian follicles while increasing the rate of follicular atresia in tilapia [60]. Treatment of follicles with melanocortin antagonist had contrasting effects. ASIP had no effect on stimulated E2 secretion, yet lower doses of SHU9119 stimulated hCG-induced E2 secretion, whereas higher doses dose-dependently inhibited hCG-induced E2 secretion. In addition, both endogenous (Asip) and synthetic competitive antagonist (SHU9119) severely stimulated basal E2 secretion. Previous results showing that that *asip1* overexpression in a zebrafish increases egg production, yet they have a smaller yolk diameter than eggs from a wild type zebrafish, which suggests variations in the vitellogenin uptake and processing into yolk proteins between both lines.

In mammals, ASIP competes exclusively with the melanocortin agonist at Mc1r and Mc4r, which is why we prioritized in situ hybridization studies to these receptor subtypes. SHU9119 binds all MCRs, which indicates that each antagonist could work via various receptors. However, previous studies have demonstrated that ASIP can antagonize the MTII binding in all Mcrs (except Mc2r) in zebrafish [17]. In contrast, SHU9119 strongly competes with MTII at sea bass (sb) Mc4r [45], yet functions as a partial agonist at sbMc1r [61] and sbMc5r [62], although the competitive antagonism remains unevaluated. Undoubtedly, further thorough pharmacological profiles of Mcrs are required in order to reach a sound conclusion. Yet, differential effects on E2 secretion suggest that SHU9119 effects may be mediated by competitive antagonism (as the agonist such as MTII has no effects on E2 secretion). Competitive antagonists function exclusively by binding competition to the receptor with no effect on its own, thus suggesting that MSH peptides could modulate the Mcr signaling in a constitutive manner and providing an explanation for the absence of effects of MTII, while supporting a role for MSH peptides as paracrine regulators.

In contrast, it can be unprecedentedly indicated that ACTH severely stimulated T secretion in testicular cells at similar levels as those recorded for hCG yet no effect of MTII on either basal or hCG-stimulated levels was observed. Cortisol can promote in vitro spermatogonial proliferation in an androgen independent manner in zebrafish (see above). Therefore, ACTH effects on steroid secretion should be independent of cortisol secretion in zebrafish testes, and potentially mediated by binding to the Mc4r/Mrap2a complex [4,13] or Mc5r [4]. Since no expression was localized in the Leydig cells, this effect should be mediated through an indirect pathway. The effects of endogenous and chemical antagonists were ambiguous, as strictly the highest doses of ASIP were able to potentiate stimulatory effects of hCG with no effect of SHU9119. In summary and regardless of the receptor signaling and pathway, our results support the participation of the melanocortin system in the testis physiology.

## 5. Conclusions

In conclusion, it can be shown that the key components of the melanocortin system are expressed in both zebrafish testes and ovaries. Both ACTH and MSH peptides inhibit ovarian steroid synthesis whereas endogenous and chemical antagonists promote basal E2 secretion. On the contrary, ACTH, except MSH-like peptides, promote T synthesis in testicular cells with no major effects of melanocortin antagonists. Melanocortin effects can be mediated directly by modulation of melanocortin receptor signaling, expressed in both ovaries and testes. While ACTH inhibitory effects on hCG-stimulated E2 secretion seem to be related to the deleterious effects of stress on the female reproductive axis, the physiological involvement of MSH peptides on ovarian gametogenesis as well as the stimulatory effects of ACTH on T testicular synthesis remain unknown. Further studies would be required in order to unravel the physiological significance of these results.

## Figures and Tables

**Table 1:** Primers and probes used for gene expression studies by quantitative PCR.

Gene	References	Sequences (5′ → 3′)	Annealing T (°C)	Concentration
*mc1r*	[15]	F:TCCCACAAACCCTTACTGCAAG	57	250 nM
		R:TACACTGCAAAGCACCACGAAC		
*mc2r*	[4]	F:CCTGTTAGCACGCCATCATG	60	300 nM
		R:AGGCCGCTTTTCCTGTGTT		
		P:[6FAM]AAACCGAATCGCGTCTATGCCTGGT[TAM]		
*mc3r*	Present work	F:TGTGATTGACCCGCTCATCTATG	65	200 nM
		R:TCTTCCCACATCCATTCTCAGTTC		
*mc4r*	[4]	F:GCCTCGCTCTACGTCCACAT	60	300 nM
		R:CGGCGATCCGTTTCATG		
		P:[6FAM]TTCCTTCTAGCCCGGCTG[TAM]		
*mc5ra*	Present work	F:ATCATCTGCTGCTATAGTCTGA	57	200 nM
		R:ATCCACCGATCATATCCATCT		
*mc5rb*	Present work	F:CGCACTCAGGAGCCAAGAGATG	65	200 nM
		R:AGTTCCTCCAGGCACCTTCTTC		
*mrap1*	[4]	F:CTTCTTCTTGATTTTGTCACTTATTTCAC	60	300 nM
		R:TCTTTACTGAGATGATGCATAACCTTTC		
		P:[6FAM]CCCTCGAGTCAAAAAATCCGGTTTGC[TAM]		
*mrap2a*	[4]	F:AGAGCCGCCACTGATGCT	60	300 nM
		R:CCACTTGGCCTCTGGAGTTG		
		P:[6FAM]CTCTCACCCATGGACGATCAGGCA[TAM]		
*mrap2b*	[4]	F:TTGGCTGTGAGCTGGAAGTG	60	300 nM
		R:TGAAAGAGGGAACGTGATTGG		
		P:[6FAM]CATTTTCTCTGCCACCGCTGCCTG[TAM]		
*pomca*	[16]	F:AAATGACCCATTTCCGCTGGAG	60	250 nM
		R:CCCACCTTCGTTTCTATGCATG		
*pomcb*	[16]	F:AAACAACGGGAAGTATCGCATG	60	250 nM
		R:TCTGTGAACTGCTGTCCATTGC		
*asip*	[17]	F:CTGTGGGCGAGCTGCAAGAG	60	250 nM
		R:GCAGGGCTCCATAAACAGGAT		
*agrp1*	[16]	F:GTGAATGTTGTGGTGATGG	60	250 nM
		R:TTCTTCTGCTGAGTTTATTTC		
*agrp2*	[16]	F:GCTCTTCATCTGCTTGTTCTT	55	250 nM
		R:CTCCTGATTCCACACTCCT		
*18s*	[15]	F:TGCATGGCCGTTCTTAGTTG	60	150 nM
		R:AGTCTCGTTCGTTATCGGAATGA		

Sequences are shown for the forward (F) and reverse (R) primers and the TaqMan hydrolysis probe (P).

**Table 2:** Melanocortin receptor expression in different organ/tissues of several specie.

Mammals				Fish and Other Vertebrates		
Receptor	Organism	Organ/Tissue	Reference	Organism	Organ/Tissue	Reference
MC1R	Human	Leydig and corpus luteum cells	[24]	Platyfish, medaka and orange-spotted grouper	Testes and ovaries	[25,26]
				Zebrafish	Testes	[2]
MC2R	Mouse	Testes/Ovaries	[38]	Zebrafish, rainbow trout and carp		[3,14,27,28]
				Sea bass	Testes	[29]
MC3R	Human	Testes and ovaries	[41]			
	Mouse	Hypothalamus-pituitary-gonad axis	[33]			
	Mice	Fetal testes	[34]	Zebrafish, hibernating cavefish, rainbow trout and topmouth culter	Testes and ovaries	[30,31,32]
	Bovine	Granulosa and corpus luteum cells	[42]			
MC4R	Bovine	Antral follicle	[42]	Birds	Testes and ovaries	[39]
	Mice	Ovaries	[43]	Goldfish, sea bass, spotted scat, medaka	Testes and ovaries	[22,44,45,46]
	Mice	Fetal testes	[34]	Zebrafish	Testes and ovaries	[4,36]
MC5R	Mice	Testes	[37]	Birds	Testes and ovaries	[39]
	Fetal mice	Spermatogonia and mesenchymal cells	[38]	Rainbow trout and zebrafish	Ovaries	[36,40]
				Zebrafish	Testes and ovaries	Present work

## Data Availability

The data that support the findings of this study are available from the corresponding author, J.M.C.-R. and A.R., upon reasonable request.
